# The Heavy Chain 4F2hc Modulates the Substrate Affinity and Specificity of the Light Chains LAT1 and LAT2

**DOI:** 10.3390/ijms21207573

**Published:** 2020-10-14

**Authors:** Satish Kantipudi, Jean-Marc Jeckelmann, Zöhre Ucurum, Patrick D. Bosshart, Dimitrios Fotiadis

**Affiliations:** Institute of Biochemistry and Molecular Medicine, and Swiss National Centre of Competence in Research (NCCR) TransCure, University of Bern, CH-3012 Bern, Switzerland; satish.kantipudi@ibmm.unibe.ch (S.K.); jean-marc.jeckelmann@ibmm.unibe.ch (J.-M.J.); zoehre.ucurum@ibmm.unibe.ch (Z.U.); patrick.bosshart@ibmm.unibe.ch (P.D.B.)

**Keywords:** 4F2hc, amino acid transporter, heterodimeric amino acid transporter, LAT1, LAT2, solute carrier 3 family, solute carrier 7 family

## Abstract

The human L-type amino acid transporters LAT1 and LAT2 mediate the transport of amino acids and amino acid derivatives across plasma membranes in a sodium-independent, obligatory antiport mode. In mammalian cells, LAT1 and LAT2 associate with the type-II membrane N-glycoprotein 4F2hc to form heteromeric amino acid transporters (HATs). The glycosylated ancillary protein 4F2hc is known to be important for successful trafficking of the unglycosylated transporters to the plasma membrane. The heavy (i.e., 4F2hc) and light (i.e., LAT1 and LAT2) chains belong to the solute carrier (SLC) families SLC3 and SLC7, and are covalently linked by a conserved disulfide bridge. Overexpression, absence, or malfunction of certain HATs is associated with human diseases and HATs are therefore considered therapeutic targets. Here, we present a comparative, functional characterization of the HATs 4F2hc-LAT1 and 4F2hc-LAT2, and their light chains LAT1 and LAT2. For this purpose, the HATs and the light chains were expressed in the methylotrophic yeast *Pichia pastoris* and a radiolabel transport assay was established. Importantly and in contrast to mammalian cells, *P. pastoris* has proven useful as eukaryotic expression system to successfully express human LAT1 and LAT2 in the plasma membrane without the requirement of co-expressed trafficking chaperone 4F2hc. Our results show a novel function of the heavy chain 4F2hc that impacts transport by modulating the substrate affinity and specificity of corresponding LATs. In addition, the presented data confirm that the light chains LAT1 and LAT2 constitute the substrate-transporting subunits of the HATs, and that light chains are also functional in the absence of the ancillary protein 4F2hc.

## 1. Introduction

Amino acids are essential biomolecules, which are involved in cellular processes ranging from energy production to protein synthesis and signaling. Membrane proteins belonging to different solute carrier (SLC) families mediate the transport of amino acids and their derivatives across biological membranes [1]. Among these transporter families, the SLC7 family consists of 15 genes [2], which encode amino acid transporters that belong to the amino acid, polyamine and organocation (APC) superfamily of transporters (transport classification (TC) system No. 2.A.3; http://www.tcdb.org) [3]. The SLC7 family consists of two subgroups: the cationic amino acid transporters (CATs; *SLC7A1-A4* and *SLC7A14*) and the glycoprotein-associated L-type amino acid transporters (LATs; *SLC7A5-A11, Slc7a12, SLC7A13,* and *Slc7a15*) [2]. CATs are N-glycosylated, while LATs are not. In contrast to CATs, LATs associate with type-II membrane N-glycoproteins, which belong to the SLC3 family, e.g., 4F2hc (SLC3A2; CD98) and rBAT (SLC3A1), to form heterodimeric amino acid transporters (HATs). These ancillary proteins, also called heavy chains, contain an intracellular, N-terminal domain, a single transmembrane α-helix, and a large extracellular C-terminal domain. The atomic structure of the extracellular domain of the heavy chain 4F2hc is similar to the structure of bacterial glucosidases, but does not possess glucosidase activity [4]. The ancillary glycoproteins 4F2hc and rBAT, which are covalently linked to corresponding LATs, i.e., the light chains of HATs, by conserved disulfide bridges are essential for the correct trafficking of the heterodimer to the plasma membrane in mammalian cells [2,5,6].

HATs have major impacts on human health being implicated in several human diseases such as aminoacidurias (cystinuria and lysinuric protein intolerance), tumor cell growth, glioma invasion, Kaposi’s sarcoma-associated herpesvirus infection, and cocaine relapse [2,5,7]. 4F2hc-LAT1 and 4F2hc-LAT2 are expressed in different tissues, e.g., LAT1 (SLC7A5) in brain, ovary, testis, placenta, spleen, colon, blood-brain barrier, fetal liver, activated lymphocytes, tumor cells and LAT2 (SLC7A8) in small intestine, kidney, lung, heart, spleen, liver, brain, placenta, prostate, ovary, fetal liver, testis, skeletal muscle [2]. They mediate sodium-independent obligatory exchange of substrates across cell membranes with a 1:1 stoichiometry [2,5]. Predominant substrates of 4F2hc-LAT1 are large neutral L-amino acids [8], L-DOPA [9], and the thyroid hormones T_3_ and T_4_ [10,11]. 4F2hc-LAT2 has specificity toward neutral L-amino acids including small ones [8,12,13], and T_3_ and T_4_ [11]. 4F2hc-LAT1 is a target for cancer diagnosis and treatment [1,2,14,15,16]. In several cancer cells, 4F2hc-LAT1 is overexpressed mediating increased uptake of L-leucine. Relative high concentrations in L-leucine result in increased mammalian target of rapamycin (mTOR) activation [17], which supports growth and survival of cancer cells [18]. Because of its localization in the blood-brain barrier, 4F2hc-LAT1 is a promising transport system, which is also utilized to shuttle drugs and prodrugs into the brain [19]. Recently, it was suggested that lack or defects in LAT2 are implicated in age-related hearing loss [20] and cataract formation [21].

Cryo- and negative stain-electron microscopy (EM) elucidated the supramolecular organization and structures of selected HATs, i.e., of 4F2hc-LAT1 [22,23,24], 4F2hc-LAT2 [25,26,27,28], and rBAT-b^0,+^AT [29]. Furthermore, the high-resolution cryo-EM structures of 4F2hc-LAT1 [22,23] and rBAT-b^0,+^AT [29] provided detailed insights into the interactions between the ancillary glycoproteins and the corresponding membrane transporters at the molecular level.

The methylotrophic yeast *Pichia pastoris* has been successfully used for the overexpression of eukaryotic membrane proteins [30,31] and for large-scale production of recombinant soluble and membrane proteins at high cell densities [32]. In an overexpression screening campaign using *P. pastoris*, the human HAT 4F2hc-LAT2 was successfully identified as a promising candidate for milligram protein production [33]. Expression in *P. pastoris* resulted in functional recombinant human 4F2hc-LAT2 containing the conserved disulfide bridge between light and heavy chain [25,26,33]. Importantly, when LAT2 is expressed in *P. Pastoris*, the recombinant protein is properly folded, correctly trafficked to the plasma membrane and functional even in the absence of co-expressed 4F2hc [25]. In contrast, surface expression of LAT2 in the absence of its ancillary glycoprotein is severely impaired in mammalian cells [6]. This important difference makes the Pichia expression system special, and gives the opportunity to study LATs alone, e.g., LAT1 and LAT2, and to explore possible effects of the ancillary glycoprotein 4F2hc on their transport function. To this aim, we expressed the HATs 4F2hc-LAT1 and 4F2hc-LAT2, and the LATs LAT1 and LAT2 in the methylotrophic yeast *P. pastoris*, and characterized and compared their L-leucine transport kinetics and amino acid specificities using [^3^H]L-leucine-based uptake and competition assays. We found that the heavy chain 4F2hc modulates the substrate affinity and specificity of LATs. In addition, the presented data confirm that the light chains LAT1 and LAT2 constitute the substrate-transporting components of the HATs and that these two light chains are also functional in the absence of 4F2hc. Thus, *P. pastoris* has proven useful as eukaryotic system to express and characterize the transport function of the human light chains LAT1 and LAT2 in the absence of co-expressed heavy chain/ancillary protein 4F2hc.

## 2. Results and Discussion

**Figure 1:**
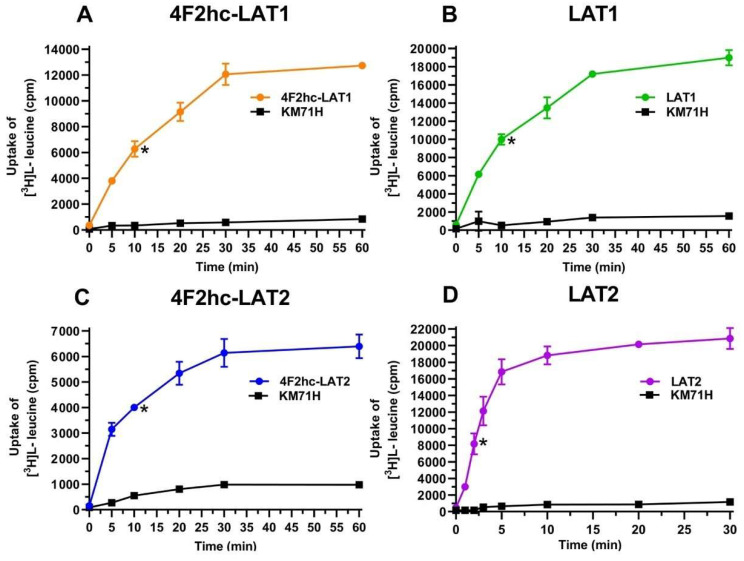
Time-dependent uptake of 100 nM [^3^H]L-leucine into *P. pastoris* KM71H cells expressing 4F2hc-LAT1 ((**A**); orange), LAT1 ((**B**); green), 4F2hc-LAT2 ((**C**); blue), or LAT2 ((**D**); violet). [^3^H]L-leucine uptake into untransformed *P. pastoris* KM71H cells is shown in black. In all cases, a saturation of the transport process was observed. Time-course experiments show clear LAT-specific transport, which is much higher than the uptake into untransformed host cells. Uptake assay times of 10 min for 4F2hc-LAT1, LAT1, and 4F2hc-LAT2, and of 2 min for LAT2 were chosen for all subsequently presented experiments (time points indicated by *). Data points are represented as mean with SD from a representative triplicate experiment. If not visible, error bars are smaller than symbols.

The human HATs: 4F2hc-LAT1 and 4F2hc-LAT2, and LATs: LAT1 and LAT2 were expressed in the methylotrophic yeast *Pichia pastoris*. Western blot analysis indicated expression of the corresponding HATs and LATs (Figure S1). Transport activities were determined by measuring the uptake of [^3^H]L-leucine into *P. pastoris* cells expressing the corresponding HAT or LAT. Time-course experiments showed clear HAT- and LAT-specific transport activities, which were much higher than the [^3^H]L-leucine uptake into untransformed host cells (Figure 1).

**Figure 2:**
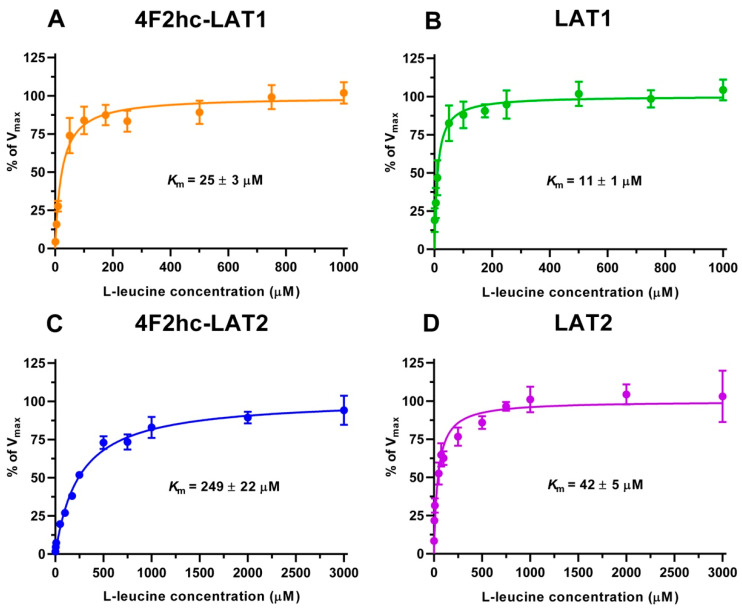
Kinetic analysis of [^3^H]L-leucine uptake into *P. pastoris* KM71H cells expressing 4F2hc-LAT1 ((**A**); orange), LAT1 ((**B**); green), 4F2hc-LAT2 ((**C**); blue) or LAT2 ((**D**); violet). Data points represent means with SD of at least nine [^3^H]L-leucine uptake replicates from three independent experiments normalized to V_max_ (100%). *K*_m_ values were obtained by fitting a Michaelis–Menten kinetics model curve to data points.

We determined the half maximal inhibitory concentrations (IC_50_s) of L-leucine by homologues competition for all constructs using the obtained time points, i.e., 10 min (4F2hc-LAT1, LAT1, 4F2hc-LAT2) and 2 min (LAT2) (Figure S2). These IC_50_s (Figure S2) gave first impressions of the affinities of the HATs and LATs for L-leucine. HAT- and LAT-mediated transport of [^3^H]L-leucine was saturable and followed Michaelis–Menten kinetics with *K*_m_ values of 25 µM (4F2hc-LAT1), 11 µM (LAT1), 249 µM (4F2hc-LAT2), and 42 µM (LAT2) (Figure 2). As reflected from this data, the impact of the heavy chain 4F2hc on the affinity of the light chain for L-leucine was most pronounced for LAT2, where the *K*_m_ value increases almost six-fold upon association with 4F2hc. The measured *K*_m_s of L-leucine for the two HATs were comparable with values from previous publications, i.e., 18 µM [34] and 20 µM [35] for 4F2hc-LAT1, and 220 µM for 4F2hc-LAT2 [13]. Recently, a study using proteoliposomes showed that human LAT1 has no transport activity in the absence of 4F2hc and concluded that the ancillary protein is essential for the transport activity of the complex [23]. In contrast, we demonstrate that LAT1 and LAT2 are able to transport [^3^H]L-leucine in the absence of the heavy chain 4F2hc. This finding is further supported by studies, in which LAT1 was reconstituted into liposomes, and transport activity of this light chain was shown in the absence of the heavy chain 4F2hc [36,37].

**Figure 3:**
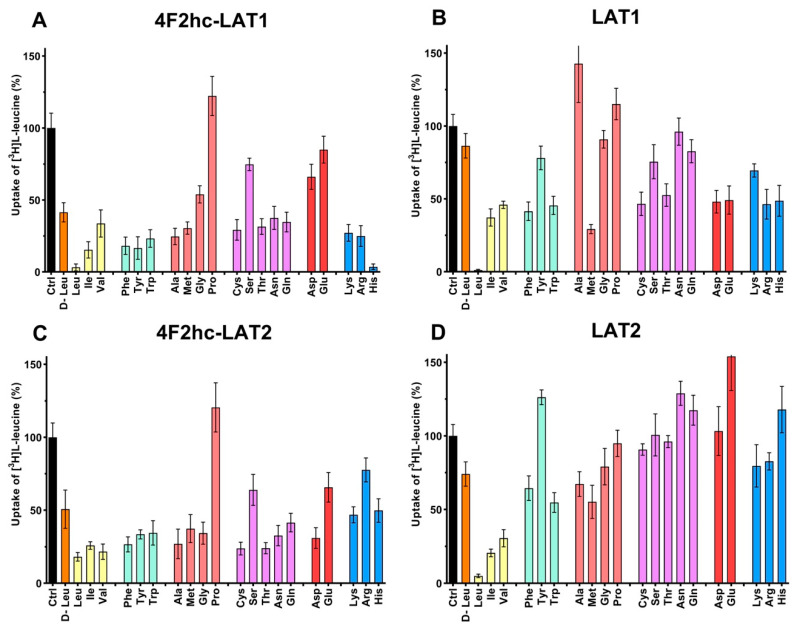
Determination of the substrate specificity of 4F2hc-LAT1 (**A**), LAT1 (**B**), 4F2hc-LAT2 (**C**), and LAT2 (**D**) by 100 nM [^3^H]L-leucine uptake competition assay. With the exception of D-leucine, proteogenic L-amino acids were used as competitors. Competitor concentrations of 250 µM ((**A**); 4F2hc-LAT1), 100 µM ((**B**); LAT1), 2500 µM ((**C**); 4F2hc-LAT2), and 500 µM ((**D**); LAT2) were used. These concentrations correspond to about ten times the determined L-leucine *K*_m_ values of the corresponding transporters (Figure 2). Residual uptake in the presence of competitor was normalized with respect to control samples without competitor (Ctrl). The amino acids are abbreviated using their three-letter-code. Means with SD from normalized data of three independent experiments, each at least in triplicate are shown. If not visible, error bars are smaller than symbols.

**Figure 4:**
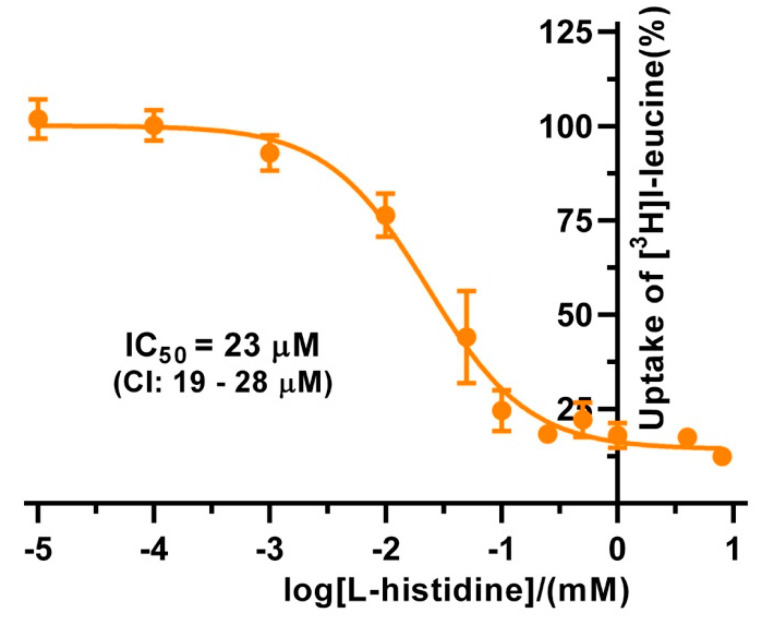
IC_50_ determination of L-histidine for human 4F2hc-LAT1 transporter. The determined IC_50_ value for 4F2hc-LAT1 transporter is 23 µM (95% confidence interval (CI): 19–28 µM). For data analysis, the signal of the untransformed *P. pastoris* KM71H was subtracted from 4F2hc-LAT1 transporter. Cpm values of each experiment were normalized with respect to the determined upper plateau value, i.e., the fitted upper plateau value corresponds to 100%. A sigmoidal model curve (orange) was fitted to the net transport signals to obtain the IC_50_. Data points represent means with SD from normalized data of three independent experiments, each at least in triplicate are shown. If not visible, error bars are smaller than symbols.

The substrate specificities of 4F2hc-LAT1 and 4F2hc-LAT2, and LAT1 and LAT2 were determined by measuring the ability of proteinogenic amino acids and D-leucine at concentrations of about ten times *K*_m_ to compete with [^3^H]L-leucine uptake (Figure 3). 4F2hc-LAT1 showed a relatively broad substrate specificity with highest for L-leucine and L-histidine, in line with previous reports [34,35,36]. The specificity for D-leucine was significantly lower compared to its L-isomer indicating stereospecificity of 4F2hc-LAT1 (Figure 3). Having established an [^3^H]L-leucine-based uptake assay and for comparison with kinetic values in the literature, we determined the half maximal inhibition concentration (IC_50_) for L-histidine to 23 µM using *P. pastoris* cells expressing human 4F2hc-LAT1 (Figure 4). This IC_50_ is comparable to previously reported *K*_m_ values for L-histidine, i.e., 12.7 µM [35] and 24.6 µM [36]. Competition data clearly showed that this HAT, 4F2hc-LAT1, has no considerable affinity (i.e., more than 50% residual [^3^H]L-leucine uptake; Figure 3A) for glycine, L-proline, L-serine and the negatively charged amino acids L-aspartate and L-glutamate. The specificity for most tested amino acids decreased when [^3^H]L-leucine transport competition was studied for the light chain LAT1 alone (Figure 3B). The stereospecificity with respect to L-leucine increased significantly compared to 4F2hc-LAT1, i.e., almost no observed inhibition of [^3^H]L-leucine transport by D-leucine (Figure 3B). L-alanine, which was competing with HAT-mediated L-leucine transport, was not recognized by LAT1 at all. In summary, LAT1 is highly specific for L-leucine and has a modest affinity for other amino acids (Figure 3B). Interestingly, the effect of competition for L-histidine in LAT1 is significantly decreased in the absence of the heavy chain 4F2hc (Figure 3A,B), which suggests a markedly lower affinity for this amino acid compared to the heterodimer. As observed for 4F2hc-LAT1, the HAT 4F2hc-LAT2 has also a relatively broad substrate specificity (Figure 3C). Most amino acids with the exception of L-proline, L-serine, L-glutamate, and the positively charged amino acids L-lysine, L-arginine, and L-histidine reduced the residual [^3^H]L-leucine uptake below 45%.

In contrast to 4F2hc-LAT1, specificities of 4F2hc-LAT2 for the branched chain amino acids (BCAA) L-leucine, L-isoleucine, and L-valine were comparable (Figure 3C). Again, in the absence of the heavy chain 4F2hc the competition pattern changed significantly (Figure 3D). As observed for LAT1, L-leucine showed the strongest reduction of LAT2-mediated [^3^H]L-leucine uptake in contrast to its D-isomer, reflecting stereospecificity of the transporter (Figure 3D). Only L-isoleucine and L-valine also reduced the residual [^3^H]L-leucine uptake below 35%, which shows that LAT2 has a preferred affinity for BCAA. The strongest increase in competition associated with co-expression of the heavy chain 4F2hc is observed for L-tyrosine, L-cysteine, L-threonine, L-asparagine, L-glutamine, and L-aspartate. In general, association of the light chain LAT2 with the heavy chain 4F2hc expands the substrate specificity of the HAT compared to the BCAA-specific light chain LAT2 alone. Interestingly, the stereospecificity for leucine of the light chains LAT1 and LAT2 is more pronounced in the absence of the heavy chain 4F2hc (Figure 4).

## 3. Conclusions

We have shown and confirmed that the light chains LAT1 and LAT2 are the substrate-transporting subunits of the corresponding HATs 4F2hc-LAT1 and 4F2hc-LAT2, and that 4F2hc is not essential for the transport activity of the corresponding LATs. LAT1 and LAT2 have relatively high specificities for L-leucine, and modest specificities for other amino acids. That the ancillary protein 4F2hc is responsible for chaperoning the trafficking of light chains (i.e., LATs) to the plasma membrane of mammalian cells is a well-accepted and described concept. Our comparative transporter study revealed a novel function of 4F2hc, i.e., upon association of this ancillary protein with LAT1 and LAT2, the substrate affinity and specificity of these light subunits is modulated by significantly broadening their substrate specificities. The methylotrophic yeast *P. pastoris* has proven useful for the here-presented comparative transporter study. In contrast to mammalian cells, the substrate-transporting subunits LAT1 and LAT2 could be successfully expressed functional in Pichia in the absence of their ancillary N-glycoprotein 4F2hc. Therefore, this eukaryotic expression system, which also allows post-translational modifications such as glycosylation and disulfide bridge formation, opens the possibility to investigate the influence of pharmacologically relevant compounds on the function of heteromeric complexes and their transporters alone.

## 4. Materials and Methods

### 4.1. Cloning of Human 4F2hc-LAT1, 4F2hc-LAT2, LAT1, and LAT2

The making of the 4F2hc-LAT2 and LAT2 expression constructs, i.e., pPICZB-4F2hc-LAT2 and pPICZB-LAT2 in the pPICZB vector (Thermo Fisher Scientific, Waltham, MA, USA), and of the *Pichia pastoris* clones expressing human 4F2hc-LAT2 or LAT2 was described in detail previously [33]. These same two Pichia clones expressing 4F2hc-LAT2 and LAT2 were used in the here-presented study. We generated a pPICZB-based expression construct for human 4F2hc-LAT1 (pPICZB-4F2hc-LAT1) as described for 4F2hc-LAT2 [33], but using the cDNA of the light chain LAT1 (UniProt ID code Q01650) instead of LAT2. For the LAT1 construct, the human gene (UniProt ID code Q01650) was synthesized codon-optimized for expression in the methylotrophic yeast *Pichia pastoris* with 5′-HindIII and 3′-XhoI restriction sites (GenScript). In contrast to this codon-optimized *LAT1* gene, the previously mentioned constructs were generated from cDNA. The *LAT1* gene was ligated into the vector pZUDFPICZ-10His3C using 5′-HindIII and 3′-XhoI restriction sites yielding the construct pZUDFPICZ-10His3C-LAT1. pZUDFPICZ-10His3C is a modified version of the pPICZB plasmid (Thermo Fisher Scientific), which has been modified as follows. First, the single HindIII restriction site of pPICZB was removed by site-directed mutagenesis using the primer (5′-3′) TGG TTC CAA TTG ACA AAC TTT TGA TTT TAA CGA. Then, an XbaI restriction site was introduced after the polyhistidine tag of the modified pPICZB plasmid using the primer (5′-3′) ATC ATC ATC ATC ATC ATT CTA GAT GAG TTT GTA GCC TTA GA. Both mutagenesis reactions were performed using the QuikChange site-directed mutagenesis kit (Agilent Technologies, Santa Clara, CA, USA). Finally, the region of the modified plasmid between the unique EcoRI restriction site and the newly created XbaI restriction site was replaced by the synthetic polynucleotide (5′-3′) GAA TTC **ACC ATG G**CA CAT CAT CAT CAT CAT CAT CAT CAT CAC CAC GAG CTC CTT GAG GTC CTT TTT CAG GGT CCT AAG CTT GCG GCC GCC CTC GAG TCT AGA. This results in the new expression plasmid pZUDFPICZ-10His3C with a Kozak sequence (in bold), an N-terminal decahistidine-tag (His-tag) followed by a human rhinovirus 3C (HRV3C) protease cleavage site and a multicloning site (HindIII, NotI, XhoI, and XbaI).

### 4.2. Pichia Pastoris Culture and Expression

Electrocompetent *P. pastoris* strain KM71H cells (Thermo Fisher Scientific) were transformed with PmeI-linearized human 4F2hc-LAT1 and LAT1 plasmids by electroporation using a Gene Pulser II system (Bio-Rad, Hercules, CA, USA) and the settings 1.5 kV, 200 Ω, and 25 µF. To select for plasmid integration, transformed *P. pastoris* cells were plated on YPD-agar plates (1% (*v*/*v*) bacto yeast extract (BD Biosciences, Franklin Lakes, NJ, USA), 2% (*w*/*v*) peptone (Condalab, Madrid, Spain), 2% (*w*/*v*) dextrose (Sigma, St. Louis, MO, USA), 2% (*w*/*v*) agar (BD Biosciences) supplemented with 200 µg/mL zeocin (InvivoGen, San Diego, CA, USA) and incubated for 2–3 days at 30°C. Colonies, which grew in the presence of 200 µg/mL zeocin, were streaked on new YPD-agar plates containing increasing zeocin concentrations (500, 1000, 2000, and 4000 µg/mL). Each plate was incubated for 2–3 days at 30 °C. To screen for clones, which grew in the presence of 4000 µg/mL zeocin with high transport activity, individual colonies were cultivated under expression conditions and their transport activities were assessed. Clones of corresponding constructs showing the highest uptake of [^3^H]L-leucine were selected for further experiments and verified for correct integration by PCR. Selected clones and untransformed *P. pastoris* KM71H cells were initially inoculated in 10 mL of YPD media as a seed culture in 50 mL culture tubes and grown for 24 h at 30 °C and 300 rpm in an incubator shaker (Multitron, Infors HT, Bottmingen, Switzerland). From seed cultures, 5 mL of inoculum were added to 500 mL buffered glycerol-complex medium (BMGY; 1% (*v*/*v*) glycerol, 1% (*w*/*v*) bacto yeast extract (BD Biosciences), 2% (*w*/*v*) peptone (Condalab), 100 mM potassium phosphate pH 6.0, 1.34% (*w*/*v*) yeast nitrogen base YNB (Condalab), 4 × 10^−5^% (*w*/*v*) biotin) and grown overnight (12–14 h) to OD_600_ 4–6 at 30 °C and 200 rpm using 2 L Erlenmeyer culture flasks in an incubator shaker (Multitron, Infors HT). For protein expression, cells were pelleted by centrifugation (3000× *g*, 15 min, room temperature) and resuspended in 1/5 to 1/10 of the original culture volume (as described in the manufacturer’s manual for Mut^s^ strains; Thermo Fisher Scientific) in buffered methanol-complex medium (BMMY; buffered-complex medium with the same composition as BMGY except having a final concentration of 1% (*v*/*v*) methanol instead of 1% (*v*/*v*) glycerol) to a final cell density of OD_600_ 40. Resuspended cells (50–75 mL) were grown in 1 L Erlenmeyer culture flasks at 30 °C and 300 rpm in an incubator shaker (Multitron, Infors HT). Conditions for induction were maintained by supplementing the expression culture with methanol after 20 and 24 h to a final concentration of 1% (*v*/*v*). Cells were harvested 28 h post induction by centrifugation (3000× *g*, 15 min, room temperature). The resulting pellet was resuspended in transport buffer (150 mM choline chloride (ChCl), 1 mM MgCl_2_, 1 mM CaCl_2_, 10 mM HEPES and 10 mM Tris, pH 7.4) containing 50% (*v*/*v*) glycerol, the OD_600_ was adjusted to 40, and the cells were finally stored at −18 °C.

### 4.3. Western Blot Analysis

Immunoblotting experiments were performed using *P. pastoris* cells expressing the corresponding HAT or LAT used in the here-presented functional studies. For Western blots, ~15 mg Pichia cells expressing HATs or LATs were suspended in 1 mL of 50 mM potassium phosphate pH 7.4, 1 mM EDTA, 5% (*v*/*v*) glycerol, 2% (*w*/*v*) SDS, 5 mM oxidized glutathione and protease inhibitor (SigmaFAST^TM^ Protease Inhibitor Cocktail Tablet, EDTA Free, Sigma-Aldrich, St. Louis, MO, USA). Cells were lysed at room temperature by homogenization with 20 strokes using a glass Teflon homogenizer. This lysis procedure was repeated four more times after 30 min incubation between each pass. Insolubilized cells and debris were separated by centrifugation at 12,000× *g* (10 min, 4 °C). The supernatant was mixed with 5x non-reducing sample buffer (60 mM Tris-HCl, pH 6.8, 10% (*v*/*v*) glycerol, 2% (*w*/*v*) SDS, 0.01% (*w*/*v*) bromophenol blue) to a final concentration of 2.5x non-reducing sample buffer and separated on a 10% SDS-PAGE gel. The His-tagged transporter LAT1 was detected using a mouse anti-His5 primary antibody (Qiagen, catalog number 34660, Hilden, Germany) at a dilution of 1:3000 and a goat anti mouse IgG (H+L) horse radish peroxidase (HRP) conjugated secondary antibody (Bio-Rad, catalog number 172-1011) at a dilution of 1:3000. Strep-tagged transporters (4F2hc-LAT1, 4F2hc-LAT1, and LAT2) were detected with HRP-conjugated streptavidin (StrepMAB-Classic-HRP, IBA Lifesciences, catalog number 2-1509-001) at a dilution of 1:30,000.

### 4.4. [^3^H]L-Leucine Radioligand Transport Assay

For transport experiments, 3 mL *P. pastoris* cells at OD_600_ 40 expressing the corresponding transporter were thawed, diluted 1:50 in transport buffer, and pelleted by centrifugation (3000× *g*, 15 min, room temperature). Subsequently, the pellet was washed by resuspending in 50 mL transport buffer and pelleted again. The washing step was repeated twice. Finally, the cell pellet was resuspended in 4 mL of transport buffer and incubated for 20 min at 30 °C under agitation (300 rpm, Multitron, Infors HT). The density of the yeast suspension was adjusted with transport buffer to OD_600_ 25 (LAT1, 4F2hc-LAT2 or 4F2hc-LAT1) and 7.5 (LAT2). All transport experiments were performed in a reaction volume of 100 µL. For time course experiments, the reaction mixture contained 40 µL cell suspension and 60 µL substrate master mix (167 nM L-leucine spiked with [^3^H]L-leucine (American Radiolabeled Chemicals)) to a specific activity of 20 Ci/mmol resulting in a final L-leucine concentration of 100 nM. For the determination of the Michaelis–Menten constant (*K*_m_), the reaction mixture contained 40 µL cell suspension and 60 µL of L-leucine solution yielding final concentrations ranging from 1–3000 µM (4F2hc-LAT2 and LAT2) and 1–1000 µM (4F2hc-LAT1 and LAT1), which were spiked with [^3^H]L-leucine to a specific activity of 0.033 Ci/mmol. For L-leucine and L-histidine IC_50_ experiments, the reaction mixture contained 40 µL cell suspension, 50 µL of competitor solution at different concentrations, i.e., 0.01–8000 µM (L-histidine, 4F2hc-LAT1), 0.01–10,000 µM (L-leucine, 4F2hc-LAT1, and LAT1) and 1–10,000 µM (L-leucine, 4F2hc-LAT2, and LAT2), and 10 µL substrate master mix (1 µM L-leucine spiked with [^3^H]L-leucine (American Radiolabeled Chemicals, St. Louis, MO, USA)) to a specific activity of 20 Ci/mmol resulting in a final L-leucine concentration of 100 nM. For substrate profiling (i.e., competition experiments), the reaction mixture contained 40 µL cell suspension, 50 µL of competitor solution (i.e., the final competitor concentration was about 10× higher than the L-leucine *K*_m_ value of the respective transporter), and 10 µL substrate master mix (1 µM L-leucine spiked with [^3^H]L-leucine (American Radiolabeled Chemicals)) to a specific activity of 20 Ci/mmol resulting in a final L-leucine concentration of 100 nM. Competitors were prepared in 10% (*v*/*v*) DMSO yielding a final concentration of 0.5% in the assay. Control samples contained the same concentration of DMSO. Final OD_600_ values in uptake experiments were 10 for 4F2hc-LAT1, 4F2hc-LAT2, and LAT1, and 3 for LAT2. All transport reactions were done in 2 mL reaction tubes (Eppendorf) at 25 °C under agitation (1000 rpm, Thermomixer compact, Eppendorf, Hamburg, Germany). Transport was terminated after 10 min for 4F2hc-LAT1, 4F2hc-LAT2, or LAT, and 2 min for LAT2 by addition of 600 µL of pre-chilled transport buffer. Cells were rapidly separated from the buffer by transferring the stopped reactions on a 96-well 0.66 mm glass fiber filter plate (Corning FiltrEX, Corning, NY, USA) and vacuum filtration. Each well was washed with 2 mL of ice-cold transport buffer to remove free radioligand. The plate was then dried overnight at 37 °C and the backside was sealed with back seal (PerkinElmer, Waltham, MA, USA). The trapped radioligand was released by addition of 200 µL scintillation cocktail (MicroScint 40, PerkinElmer) to each well and the plate topside was sealed with Topseal^TM^-A Plus (PerkinElmer), followed by incubation for 30 min at 25 °C and 1000 rpm (Thermomixer compact, Eppendorf, Hamburg, Germany). Counts were measured in each well for 2 min with a scintillation counter (TopCount NXT, PerkinElmer).

### 4.5. Statistics

Experimental data points were performed at least in triplicate. For data analysis, the signal of the untransformed *P. pastoris* cells was subtracted from the transporter signal. Michaelis–Menten saturation curves were fitted into data points of independent experiments. Data points were then individually normalized using the corresponding V_max_ values (i.e., the fitted upper plateau value corresponds to 100%). Data points from corresponding concentrations were averaged and SD obtained. Finally, Michaelis–Menten saturation curves were fitted to the averaged data yielding *K*_m_ values. To determine the half maximal inhibitory concentration (IC_50_) values of heterologous (i.e., L-histidine) L-leucine transport competition, a sigmoidal model curve was fitted to the net transport signals of independent experiments. Every experimental data point was individually normalized using the corresponding upper plateau values (i.e., the fitted upper plateau value corresponds to 100%). Data points from corresponding concentrations were averaged and a sigmoidal model curve was fitted to the data in order to obtain the IC_50_ value. Prism6 (GraphPad Software) was used for data analysis.

## Supplementary Materials

Supplementary materials can be found at https://www.mdpi.com/1422-0067/21/20/7573/s1. Figure S1: Western blot analysis of SDS-solubilized *Pichia pastoris* cells overexpressing human 4F2hc-LAT1, LAT1, 4F2hc-LAT2, or LAT2; Figure S2: IC50 determination of L-leucine for human 4F2hc-LAT1 (A; orange), LAT1 (B; green), 4F2hc-LAT2 (C; blue), and LAT2 (D; violet).

## Abbreviations

LAT	L-type amino acid transporter
HAT	Heteromeric amino acid transporters
IC_50_	Half maximal inhibitory concentration
*K*_m_	Michaelis-Menten constant
SLC	Solute carrier
