# Supplementary material for: The Heavy Chain 4F2hc Modulates the Substrate Affinity and Specificity of the Light Chains LAT1 and LAT2
Source: Int J Mol Sci. 2020 Oct 14;21(20):7573. doi: 10.3390/ijms21207573 (PMC7589757; doi:10.3390/ijms21207573)
Supplement: Supplementary file 1 [file ijms-21-07573-s001.pdf]

International Journal of *Molecular Sciences*

MDPI

Supplementary Information

The Heavy Chain 4F2hc Modulates the Substrate Affinity and Specificity of the Light Chains LAT1 and LAT2

Satish Kantipudi, Jean-Marc Jeckelmann, Zöhre Ucurum, Patrick D. Bosshart and Dimitrios Fotiadis *

Institute of Biochemistry and Molecular Medicine, and Swiss National Centre of Competence in Research (NCCR) TransCure, University of Bern, CH-3012 Bern, Switzerland; satish.kantipudi@ibmm.unibe.ch (S.K.); jean-marc.jeckelmann@ibmm.unibe.ch (J.-M.J.); zoehre.ucurum@ibmm.unibe.ch (Z.U.); patrick.bosshart@ibmm.unibe.ch (P.D.B.)

* Correspondence: dimitrios.fotiadis@ibmm.unibe.ch; Tel.: +41-31-631-4103

Received: 16 August 2020; Accepted: 10 October 2020; Published: date

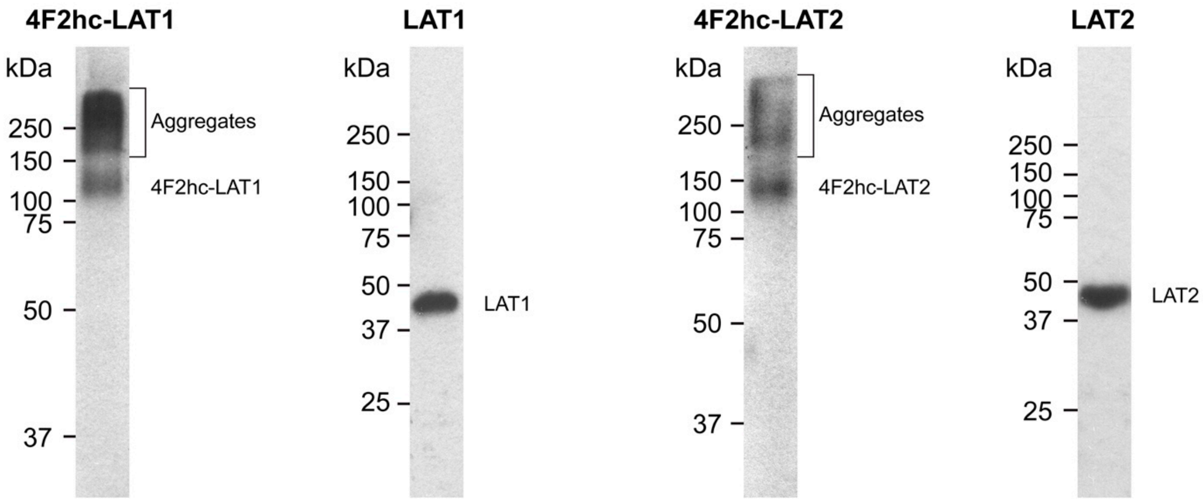

Figure S1. Western blot analysis of SDS-solubilized *Pichia pastoris* cells overexpressing human 4F2hc-LAT1, LAT1, 4F2hc-LAT2 or LAT2. For 4F2hc-LAT1 and 4F2hc-LAT2, a prominent band at ~120 kDa was observed. This molecular weight is in line with the expected mass of the heteromeric amino acid transporters (HATs) [1]. As observed previously with human 4F2hc-LAT2, HATs had to some extent the tendency to aggregate in SDS-PAGE [1]. LAT1 and LAT2 Western blots displayed a single band near the 50 kDa marker, in agreement with previously described migration properties [1]. See Materials and Methods for a detailed description of immunoblotting experiments and Figure S3 for full original images of Western blots.

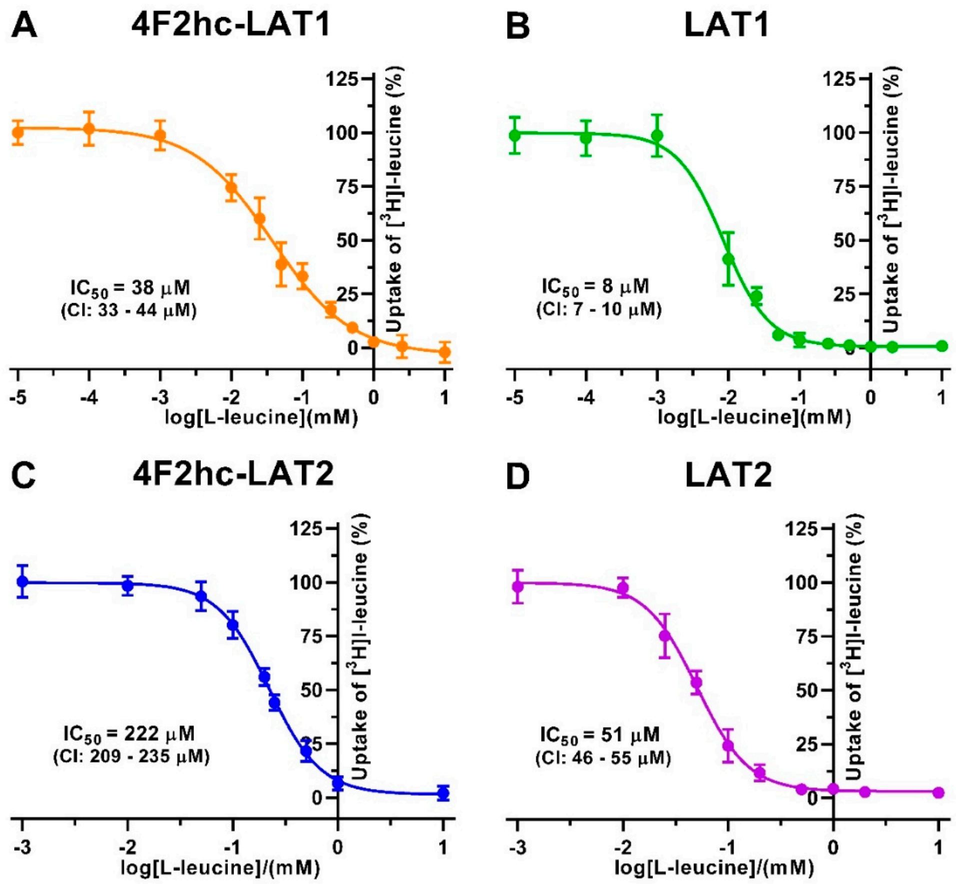

Figure S2. IC_{50} determination of L-leucine for human 4F2hc-LAT1 (**A**; orange), LAT1 (**B**; green), 4F2hc-LAT2 (**C**; blue) and LAT2 (**D**; violet). Determined IC_{50} values and 95% confidence intervals (CIs) are indicated. For data analysis, the signal of the untransformed *P. pastoris* KM71H was subtracted from all transporter signals. Cpm values of each experiment were normalized with respect to the determined upper plateau value, i.e., the fitted upper plateau value corresponds to 100%. A sigmoidal model curve was fitted to the net transport signals to obtain the IC_{50}. Data points represent means with SD from normalized data of three independent experiments, each at least in triplicate are shown. If not visible, error bars are smaller than symbols.

References

1. Costa, M.; Rosell, A.; Alvarez-Marimon, E.; Zorzano, A.; Fotiadis, D.; Palacin, M. Expression of human heteromeric amino acid transporters in the yeast *Pichia pastoris*. *Protein Expr. Purif.* **2013**, *87*, 35-40.

© 2020 by the authors. Submitted for possible open access publication under the terms and conditions of the Creative Commons Attribution (CC BY) license (http://creativecommons.org/licenses/by/4.0/).
